# Vitamin D Deficiency among Patients Visiting Outpatient Departments in a Tertiary Care Centre: A Descriptive Cross-sectional Study

**DOI:** 10.31729/jnma.7299

**Published:** 2022-04-30

**Authors:** Arun Bahadur Chand, Samir Singh, Lok Raj Bhatt, Bindu Sen, Yadav Prasad Joshi, Pramod Joshi, Lok Bahadur Shrestha, Sailendra Kumar Duwal Shrestha, Ajaya Basnet

**Affiliations:** 1 Department of Clinical Laboratory, KIST Medical College and Teaching Hospital, Gwarko, Lalitpur, Nepal; 2 Department of Dental, KIST Medical College and Teaching Hospital, Gwarko, Lalitpur, Nepal; 3 Department of Public Health, Manmohan Memorial Institute of Health Sciences, Soalteemode, Kathmandu, Nepal; 4 Department of Orthopaedics, National Academy of Medical Sciences, Mahaboudha, Kathmandu, Nepal; 5 School of Medical Sciences and The Kirby Institute, University of New South Wales, Sydney, Australia; 6 Department of Orthopaedics and Trauma Services, Nepal Armed Police Force Hospital, Halchowk, Kathmandu, Nepal; 7 Department of Medical Microbiology, Shi-Gan International College of Science and Technology, Maharajganj, Kathmandu, Nepal

**Keywords:** *deficient*, *prevalence*, *vitamin D*

## Abstract

**Introduction:**

Vitamin D deficiency is a global health issue affecting billions of people. Its deficiency results in abnormal homeostasis of calcium and phosphorous levels in an individual and results in reduced bone mineral density, which further makes them more prone to develop osteogenic disorders, such as fractures. The aim of this study is to find out the prevalence of vitamin D deficiency among patients visiting the outpatient departments in a tertiary care centre.

**Methods:**

This was a descriptive cross-sectional study done among 582 patients visiting outpatient departments in a tertiary care centre between January 1, 2019 and July 31, 2020. The study was approved by the Institutional Review Committee (Reference number: 076/077/17) of a tertiary care centre. A convenience sampling method was used. Patients' demographic detail and serum vitamin D level were determined. Data were collected retrospectively from hospital records and analysis was performed using the Statistical Package for the Social Sciences software version 17.0. Point estimate at 95% Confidence Interval was calculated along with frequency, the proportion for binary data, and mean with standard deviation for continuous data.

**Results:**

Among 582 patients enrolled in this study, 328 (56.35%) (52.32-60.38 at 95% Confidence Interval) patients were vitamin D deficient. Vitamin D deficiency was found in 238 (72.56%) females and 257 (78.35%) aged 16 to 59 years. Finally, there were 102 (31.09%) cases of vitamin D deficiency over the winter season.

**Conclusions:**

The prevalence of serum vitamin D deficiency in the current study was lower when compared to similar studies done in similar settings and similar to the prevalence from international literature.

## INTRODUCTION

Vitamin D is a fat-soluble micronutrient, which is synthesised either from the diet or through the ultraviolet irradiation of 7-dehydrocholesterol in the skin.^1^ The conversion of vitamin D into its biologically active form i.e. 1,25-dihydroxycholecalciferol,^1^ serves as a ligand for the vitamin D receptor, which helps to regulate the expression of hundreds of genes.^2^

These expressed genes owe to the maintenance of serum calcium and phosphorus homeostasis and mineralization and resorption of bone.^2,3^

Vitamin D deficiency is a global public health issue. In its deficiency, only 10-15% of dietary calcium and 50-60% of dietary phosphorus will be absorbed, which ultimately results in a decrease in bone mineralization, osteoporosis, and subsequently, the risk of other osteogenic diseases.^1,3^ In Nepal, there are limited studies concerning vitamin D deficiency among patients visiting Out Patient Department (OPD) in a tertiary care centre.

The aim of this study is to find out the prevalence of vitamin D deficiency among patients visiting OPDs in a tertiary care centre.

## METHODS

This was a descriptive cross-sectional study done among 582 patients visiting the Out Patient Departments (OPDs) in KIST Medical College and Teaching Hospital (KISTMCTH), Lalitpur, Nepal from January 1, 2019 to July 31, 2020. Ethical approval was taken from the Institutional Review Committee (IRC) of KISTMCTH (Reference number: 076/077/52). All clinically suspected vitamin D deficient patients visiting in the OPD of KISTMCTH were included in the study. The patients admitted to the Inpatient Department (IPD) who did not have complete documentation were excluded from the study. The convenience sampling method was used.

Sample size was calculated using the formula:

n = (Z^2^ × p × q) / e^2^

  = (1.96^2^ × 0.5 × 0.5) / 0.05^2^

  = 385

Where,

n = minimum required sample size
Z = 1.96 at 95% Confidence Interval (CI)
p = prevalence taken as 50% for maximum sample size calculation
q = 1-p
e = margin of error, 5%

Hence, the calculated sample size was 385. Adding 10% missing data, the final sample size was 424. However, we included 582 patients in our study.

Three mililiter blood samples were obtained from each patient and centrifuged at 3000 revolutions per minute for five minutes to separate the serum. Samples were processed in the department of clinical biochemistry of KISTMCTH by using electrochemiluminescence technology (Cobas E411 analyzer-Roche diagnostics, United States of America). Patients with vitamin D deficiency were assessed and studied for different factors such as age, gender, and season. The reference ranges for serum vitamin D levels were <20 ng/ml (deficient), 20 ng/ml to 30 ng/ml (insufficient), 30 ng/ml to 100 ng/ml (sufficient), and >100 ng/ml (toxic).^4^

Socio-demographic data of the patients were collected from the hospital record section. After complete and detailed information was obtained, it was verified, anonymized, and then entered in Microsoft Excel 2010. Data were collected retrospectively and analysis was performed using the Statistical Package for the Social Sciences software version 17.0. Point estimate at 95% Confidence Interval was calculated along with frequency, the proportion for binary data, and mean, the standard deviation for continuous data.

## RESULTS

Among 582 patients enrolled in this study, 328 (56.35%) (52.32-60.38 at 95% Confidence Interval) patients were vitamin D deficient, out of which 90 (27.44%) were males and 238 (72.56%) were female. The mean serum vitamin D levels in children was 12.7±4.7 ng/ml, adults was 20.9±13.0 ng/ml, and the elderly was 13.5±4.5 ng/ ml. A higher prevalence of vitamin D deficiency was found in patients belonging to the 16-59 years age group (Table 1).

**Table 1:** Socio-demographic details of vitamin D deficient patients (n= 328).

Variables	Vitamin D Mean±SD	Vitamin D deficient n (%)
**Age group**		
≤15 years: Children	12.7±4.7	34 (10.37)
16-59 years: Adults	20.9±13.0	257 (78.35)
≥60 years: Elderly	13.5±4.5	37 (11.28)
**Gender**		
Male	12.7±4.4	90 (27.44)
Female	13.0±4.3	238 (72.56)

**Figure 1:**
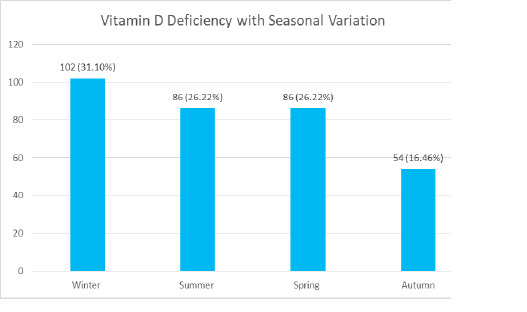
Seasonal variation of vitamin D deficiency (n= 328).

Regarding the seasonal variation of serum vitamin D deficiency, there were 102 (31.09%) cases in winter followed by 86 (26.22%) cases each in summer and spring, and 54 (16.46%) cases in autumn (Figure 1).

## DISCUSSION

Vitamin D deficiency is a global public health issue. The prevalence of vitamin D deficiency is present in one-third to one-half of healthy middle age to elderly adults.^5^ Vitamin D deficiency rickets and osteomalacia are associated with low circulating concentrations of serum vitamin D, generally less than 20 nmol/l.^6^ There are two primary sources of vitamin D for the human, namely, endogenous skin synthesis and diet.^7-9^ Inadequate sun exposure results in the high prevalence of serum vitamin D deficiency.^11^ According to the American Endocrine Society, serum vitamin D deficiency is defined as a serum vitamin D level less than 20 ng/ml, a proportional increase in parathyroid hormone, and a decrease in intestinal calcium absorption.^12^

In this study, we found that 56.35% of OPD patients had a deficiency of serum vitamin D. A similar trend of the high prevalence of serum vitamin D deficiency 73.60% among the adult population in a study done in the western region of Nepal,^13^ and another study reported 72.60% of suboptimal serum vitamin D levels in a study conducted in Saudi Arabia.^14^ A study done in Nepal found a higher prevalence of serum vitamin D deficiency among Nepalese individuals.^15^ The deficiency of serum vitamin D was found more prevalent in females in our study. Similarly, another similar study also observed a higher prevalence of serum vitamin D deficiency in females than in the male population.^16^ Several studies on vitamin D conducted in Nepal and China reported a similar pattern of vitamin D deficiency among females.^17,18^ The causes of the hypovitaminosis D in females could be due to a lack of consumption of healthy foods containing adequate amounts of vitamin D, such as dairy products, which are fortified with vitamin D.^19,20^ It has been reported that low serum vitamin D levels have been linked to disturbed menstrual periods in females too.^21^

The results of this study revealed that when comparing age groups in terms of serum vitamin D deficiency, adults had a 78.35% prevalence of serum vitamin D deficiency, followed by the elderly (11.28%) and children (10.37%). Similar findings were reported in a study by a study which revealed that 72.8% of the adults' population suffers from hypovitaminosis D.^22^ Study done in Nepal also found similar outcomes indicating that adults were more vitamin D deficient than elderly and children.^13^ The cause of this hypovitaminosis D in adults from our study could be due to a lack of Ultraviolet B (UVB) exposure, a low intake of vitamin D rich foods, and an increase in the use of sunscreen by adults.^23^

The seasonal distribution of the serum vitamin D deficient population in the current study was almost identical in all seasons except winter, which had a 31.09% prevalence. In a study showed that the serum vitamin D deficiency and the average low vitamin D levels were higher in the winter.^24^ Similarly, according to one of the study, the mean serum vitamin D level in winter was significantly lower than in summer among the general population of Estonia.^25^ This finding suggests that the season, as an environmental factor, may influence the severity of vitamin D deficiency.^26^

This was a single-centred based study with a small sample size focused on the Nepalese population; the result might not be generalizable to the global populations.

## CONCLUSIONS

The prevalence of serum vitamin D deficiency in the current study was lower than similar studies conducted in similar settings and similar to the prevalence from international literature. A higher prevalence of serum vitamin D deficiency among adult and female patients was observed.
